# Splenic Vein Thrombosis Complicating Hypertriglyceridemia-Induced Acute Pancreatitis in Diabetic Ketoacidosis: A Rare Clinical Tetrad

**DOI:** 10.7759/cureus.115258

**Published:** 2026-08-26

**Authors:** Adebola O Adetiloye, Anim Asif, Mohamed Salih, Moustafa Ismail, Olurotimi J Badero

**Affiliations:** 1 Internal Medicine, New York City Health and Hospitals/Harlem Hospital Center, New York, USA; 2 Interventional Cardiology, Iwosan Lagoon Hospital, Lagos, NGA; 3 Interventional Cardiology, Division of Cardio-Nephrology, Cardiac Renal & Vascular Associates, Jackson, USA

**Keywords:** diabetic ketoacidosis, hypertriglyceridemia, hypertriglyceridemia-induced acute pancreatitis, splenic vein thrombosis, type 2 diabetes mellitus

## Abstract

Diabetic ketoacidosis (DKA) is a life-threatening metabolic emergency that may be accompanied by severe hypertriglyceridemia (HTG), particularly in patients with poorly controlled type 1 or 2 diabetes mellitus. Marked HTG is a well-recognized cause of acute pancreatitis, while splenic vein thrombosis (SVT) is an uncommon vascular complication of pancreatic inflammation. The coexistence of these four conditions represents an exceptionally rare clinical presentation. SVT is clinically significant, as it may cause sinistral portal hypertension, gastric varices, and gastrointestinal bleeding.

A 34-year-old man with type 2 diabetes mellitus presented with abdominal pain, nausea, vomiting, and DKA. Initial evaluation revealed severe HTG and HTG-induced acute pancreatitis (HTG-AP), with CT showing a bulky, ill-defined pancreas, peripancreatic fluid, and fat stranding. He was initially treated with intravenous insulin, aggressive fluid resuscitation, and electrolyte replacement. Persistent abdominal pain prompted repeat CT on hospital day 3, which demonstrated isolated SVT with perigastric collaterals and no portal or superior mesenteric vein involvement. Therapeutic intravenous heparin was initiated, followed by transition to oral apixaban. He was discharged in stable condition on basal-bolus insulin, atorvastatin, fenofibrate, and omega-3 acid ethyl esters with multidisciplinary follow-up.

This case describes a rare clinical tetrad linking DKA, severe HTG, HTG-AP, and SVT. It highlights the importance of early recognition and prompt multidisciplinary management of both the metabolic and thrombotic complications associated with this uncommon presentation.

## Introduction

Diabetic ketoacidosis (DKA), characterized by the clinical triad of hyperglycemia, metabolic acidosis, and ketosis, remains a leading cause of morbidity and mortality across the glycemic spectrum [1]. While traditionally associated with type 1 diabetes, DKA is increasingly recognized in patients with type 2 diabetes mellitus (T2DM) during episodes of profound metabolic stress, such as infection or acute illness [1]. A critical consequence of the insulin deficiency in DKA is the inhibition of lipoprotein lipase (LPL), which triggers unrestrained peripheral lipolysis. This metabolic shift leads to a massive influx of free fatty acids to the liver, resulting in the overproduction of very-low-density lipoproteins (VLDL) and subsequent severe hypertriglyceridemia (HTG) [2].

Severe HTG is the third most common etiology of acute pancreatitis, following alcohol and gallstones. While HTG-induced acute pancreatitis (HTG-AP) typically occurs when triglyceride levels exceed 1,000 mg/dL, the concurrent acidotic state of DKA can exacerbate pancreatic injury by promoting the conversion of free fatty acids into toxic lysolecithins [3,4]. Furthermore, the intense local inflammation of the pancreas can trigger vascular complications, most notably splenic vein thrombosis (SVT). This occurs through a combination of direct endothelial injury from proximity to the inflamed pancreatic tail and a systemic prothrombotic state inherent to both DKA and acute inflammation [5]. While the "enigmatic triad" of DKA, HTG, and acute pancreatitis is well-described, the simultaneous manifestation of this triad with an acute vascular event like SVT is rare [5]. This uncommon clinical tetrad highlights the interconnected nature of metabolic dysfunction, pancreatic injury, and thrombosis, and underscores the importance of early diagnosis and individualized multidisciplinary management.

## Case presentation

A 34-year-old man with a history of T2DM, mixed hyperlipidemia, and primary hypertension presented to the emergency department with severe epigastric abdominal pain, nausea, vomiting, polyuria, and generalized weakness for 1 day. He reported poor adherence to his diabetes medications. His home medications included metformin, empagliflozin, insulin glargine, atorvastatin, lisinopril, and nifedipine. He denied alcohol use, including any history of heavy or chronic alcohol consumption. On presentation, he was alert and oriented, clinically dehydrated, without jaundice or acute respiratory distress. Vital signs were blood pressure 128/76 mmHg (normotensive), heart rate 112 beats/min (tachycardic), temperature 98.4°F (afebrile), respiratory rate 20 breaths/min, and oxygen saturation 98% on room air. Abdominal examination revealed moderate epigastric tenderness without rebound tenderness or guarding, with mild abdominal distension and decreased bowel sounds. Murphy’s sign was absent on examination. Chest examination revealed clear bilateral breath sounds with no wheezing or crackles.

**Figure 1:**
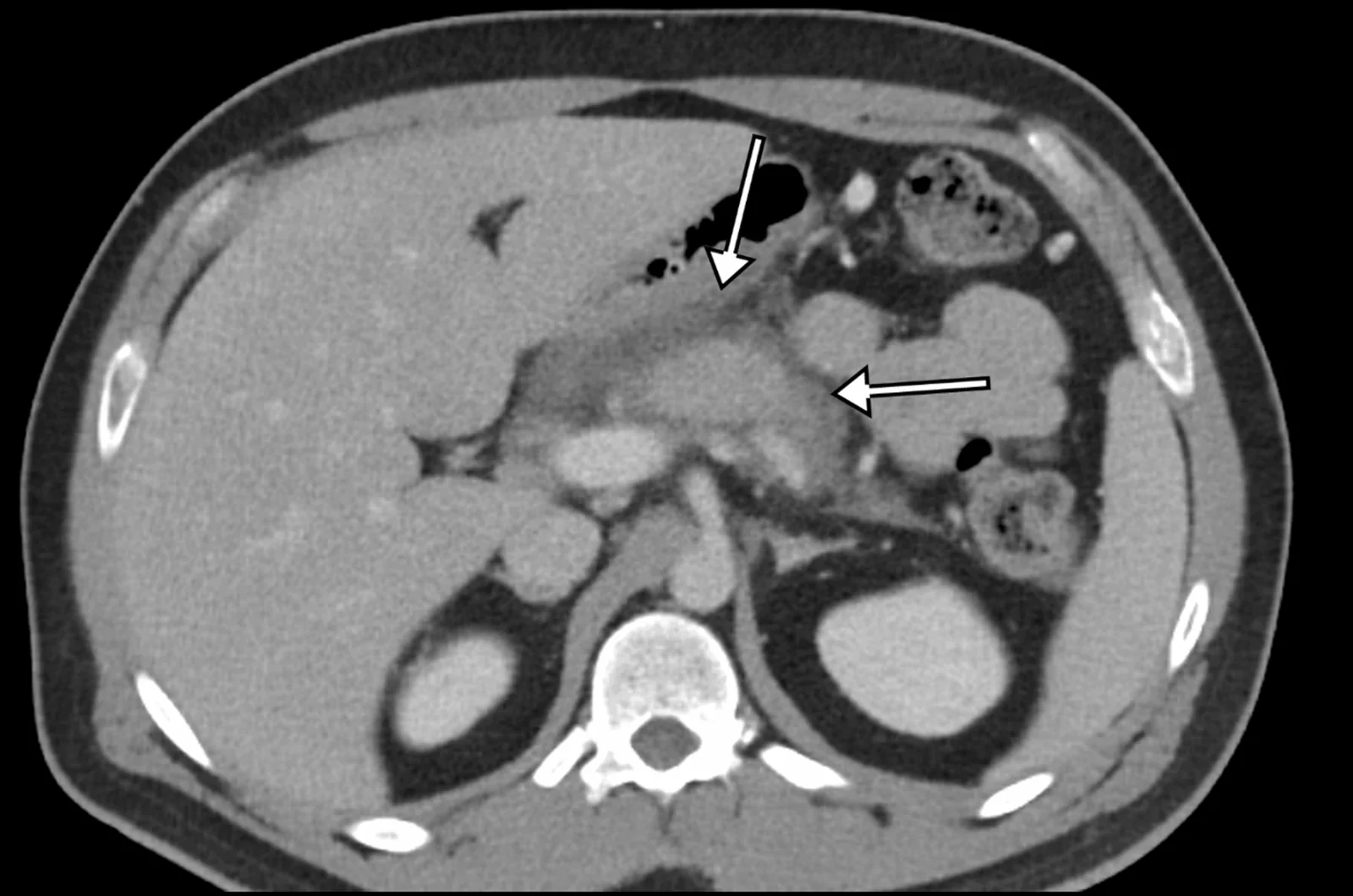
Axial contrast-enhanced computed tomography at the level of the pancreas. Arrows indicate pancreatic fluid tracking along the anterior and left margin of the bulky, ill-defined pancreas, with surrounding peripancreatic fat stranding.

Laboratory evaluation demonstrated marked hyperglycemia with glucose 470 mg/dL (reference 70-99 mg/dL), bicarbonate 14 mEq/L (reference 22-29 mEq/L), corrected sodium 130 mEq/L (reference 135-145 mEq/L), chloride 84 mEq/L (reference 98-107 mEq/L), anion gap 32 mEq/L (reference 8-12 mEq/L), and positive serum ketones, consistent with diabetic ketoacidosis. Serum triglyceride level was markedly elevated at 6362 mg/dL (reference ≤149 mg/dL) with a total cholesterol of 579 mg/dL, and serum lipase was elevated at 528 U/L (reference 13-60 U/L). Around 11 months before presentation, triglycerides were 176 mg/dL (reference ≤149 mg/dL) and LDL cholesterol was 173 mg/dL (reference <100 mg/dL). Serum beta-hydroxybutyrate was 3.75 mmol/L (reference 0.02-0.27 mmol/L), and hemoglobin A1c was greater than 14%, consistent with long-standing uncontrolled hyperglycemia. Laboratory evaluation also revealed mild leukocytosis with white blood cell count (WBC) 12.64 × 10³/µL (reference 4.8-10.8 × 10³/µL), and preserved renal function: BUN 14 mg/dL (reference: 7-18 mg/dL), creatinine 0.7 mg/dL (reference 0.7-1.3 mg/dL), and eGFR 123 mL/min/1.73 m² (reference ≥60 mL/min/1.73 m²). The admission hemoglobin of 20.5 g/dL was spuriously elevated as a result of severe lipemia. Computed tomography of the abdomen revealed findings consistent with acute pancreatitis (Figure 1).

**Figure 2:**
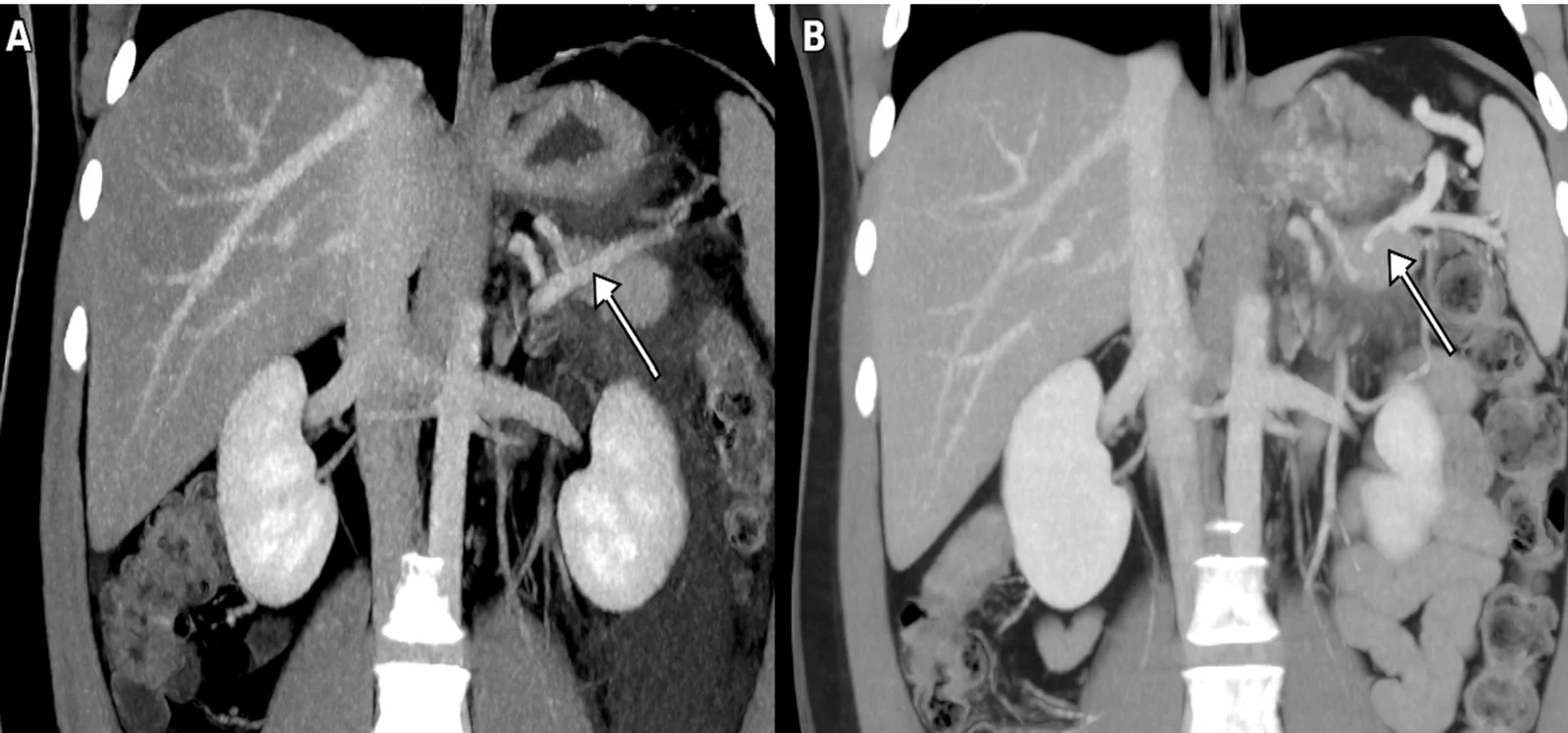
Coronal contrast-enhanced computed tomography, matched magnification. (A) Baseline: Patent, uniformly opacified splenic vein. (B) Day 3: Abrupt cutoff of the splenic vein with a non-enhancing intraluminal filling defect and hilar collateral vessels (arrow), consistent with splenic vein thrombosis.

The patient was admitted to the intensive care unit with a BISAP score of 1 (presence of SIRS) and managed with intravenous insulin infusion, aggressive intravenous fluid resuscitation, and electrolyte replacement in accordance with standard DKA protocols. Persistent abdominal pain despite initial treatment prompted repeat abdominal imaging on day 3 of admission, which subsequently demonstrated thrombosis of the splenic vein with multiple collaterals present along the stomach draining into the splenic vein without involvement of the portal or superior mesenteric veins (Figure 2).

A comprehensive thrombophilia workup, including testing for antiphospholipid syndrome, factor V Leiden mutation, prothrombin G20210A mutation, and protein C, protein S, and antithrombin deficiencies, was negative. Given the presence of SVT in the setting of acute pancreatitis, therapeutic anticoagulation with intravenous unfractionated heparin was initiated after multidisciplinary discussion. Although CT demonstrated perigastric collaterals, upper gastrointestinal endoscopy was not considered necessary before anticoagulation given the absence of clinical gastrointestinal bleeding and portal or superior mesenteric vein involvement. Within 72 hours, serum triglyceride levels had declined from 6362 mg/dL to 607 mg/dL, with a further fall to 315 mg/dL by hospital day 6. Serial laboratory values are summarized in Table 1.

**Table 1:** Serial laboratory values during hospitalization. Values outside the reference range are shown for each hospital day; a dash (-) indicates that the test was not performed on that day. NC means not calculable. ^*^The admission hemoglobin of 20.5 g/dL was flagged as a critical value but is attributable to interference from severe lipemia (concurrent MCHC 46.6 g/dL). ^†^LDL cholesterol could not be calculated by the Friedewald equation owing to triglyceride levels exceeding 400 mg/dL.

Laboratory test	Reference range	Day 1	Day 2	Day 3	Day 4	Day 5	Day 6
White blood cells	3.80-10.50 K/µL	12.64	13.23	-	7.02	-	-
Absolute neutrophils	1.80-7.40 K/µL	9.55	10.68	-	3.81	-	-
Hemoglobin	13.0-17.0 g/dL	20.5^*^	16.7	-	11	-	-
Hematocrit	39.0-50.0%	44	44.6	-	32	-	-
Platelets	150-400 K/µL	273	320	-	177	-	-
Lipase	13-60 U/L	528	-	-	-	-	-
Beta-hydroxybutyrate	0.02-0.27 mmol/L	3.75	-	-	-	-	-
Triglycerides	≤149 mg/dL	6362	2210	726	607	395	315
Total cholesterol	≤199 mg/dL	579	404	294	229	227	238
High-density lipoprotein cholesterol	≥41 mg/dL	18	19	24	17	22	22
Low-density lipoprotein cholesterol	≤99 mg/dL	NC^†^	NC^†^	NC^†^	NC^†^	133	157
Non-high-density lipoprotein cholesterol	≤129 mg/dL	561	386	270	212	205	216

Following successful reintroduction of oral intake, the patient was transitioned from intravenous insulin to a subcutaneous basal-bolus regimen. For treatment of SVT, the patient was transitioned to off-label apixaban 5 mg twice daily for six months, using the established maintenance dosing regimen for venous thromboembolism. Fenofibrate was initiated to reduce triglyceride levels and prevent recurrent HTG-induced pancreatitis, while high-intensity atorvastatin was prescribed for cardiovascular risk reduction and additional lipid-lowering. He was discharged in stable condition with multidisciplinary outpatient follow-up.

## Discussion

SVT is a recognized vascular complication of acute pancreatitis [3,5]. However, the complete tetrad of DKA, severe HTG, HTG-AP, and SVT appears to be exceptionally rare. To our knowledge, only one indexed, peer-reviewed case report has explicitly described this complete clinical constellation [5]. The association between DKA, severe HTG, and HTG-AP is well established. Insulin deficiency in DKA promotes unchecked lipolysis within adipose tissue, resulting in increased delivery of free fatty acids to the liver and enhanced hepatic synthesis and secretion of VLDL. Concurrently, reduced insulin-mediated activation of lipoprotein lipase impairs the clearance of triglyceride-rich lipoproteins, leading to profound HTG [6-9]. When triglyceride concentrations become markedly elevated, chylomicron accumulation and intrapancreatic lipolysis contribute to pancreatic ischemia, free fatty acid-mediated cytotoxicity, and inflammation, thereby precipitating acute pancreatitis [6-9]. However, the relationship may be bidirectional, as HTG-AP may itself precipitate DKA; therefore, the initiating event cannot be definitively established [6]. Acute pancreatitis may precipitate SVT through a combination of local perivenous inflammation, endothelial injury induced by pancreatic enzymes and inflammatory cytokines, extrinsic venous compression from pancreatic edema or peripancreatic fluid collections, and a systemic prothrombotic state consistent with Virchow's triad [10,11]. Pancreatitis-induced SVT occurs in approximately 14% of cases and may cause sinistral portal hypertension with gastric varices, resulting in gastrointestinal bleeding in about 12% of patients [12,13].

In addition to pancreatitis, SVT may arise from a broad range of local and systemic conditions that warrant consideration, particularly in younger patients or when the severity of pancreatitis does not fully explain the thrombotic burden. Reported causes of SVT include pancreatic and hepatobiliary malignancies, chronic liver disease with portal hypertension, myeloproliferative neoplasms, inherited or acquired thrombophilias such as antiphospholipid syndrome, factor V Leiden mutation, prothrombin gene mutation, and deficiencies of protein C, protein S, or antithrombin, as well as abdominal surgery, trauma, pancreatic inflammation with edema or pseudocysts, and systemic inflammatory or infectious disorders [14-16]. Recognition of these alternative etiologies is essential, as their presence may influence both the duration of anticoagulation and the need for targeted diagnostic evaluation.

Intravenous insulin infusion remains the cornerstone of DKA management and is an effective adjunctive therapy for HTG-AP in patients with concurrent insulin deficiency or DKA [17-19]. Insulin activates endothelial lipoprotein lipase, accelerating chylomicron clearance while suppressing hormone-sensitive lipase, thereby reducing triglyceride production and circulating free fatty acids [18,20]. The role of anticoagulation in pancreatitis-related SVT remains debated, particularly in cases of isolated involvement. Consensus statements and review of data support anticoagulation more strongly for portal or superior mesenteric vein thrombosis because of the associated risk of bowel ischemia, whereas isolated SVT lacks uniform guidance and requires individualized assessment based on thrombus extent, symptom burden, bleeding risk, and the presence of complications such as gastrointestinal varices or left-sided portal hypertension [21]. Although a recent meta-analysis supports improved venous recanalization with anticoagulation and no clear increase in major bleeding, an earlier single retrospective study did not demonstrate a significant recanalization benefit over conservative management [22,23]. In addition, use of anticoagulation has not been shown to improve survival [22]. More randomized controlled trials are needed to define the role of anticoagulation in patients with pancreatitis-related SVT. In patients with acute pancreatitis-associated SVT who are treated with anticoagulation, 3 to 6 months of therapy is recommended, with longer treatment reserved for persistent risk factors or underlying thrombophilia [24,25]. In the present case, anticoagulation with intravenous unfractionated heparin followed by transition to a direct oral anticoagulant was associated with clinical improvement.

## Conclusions

This case highlights the rare tetrad of DKA, severe HTG, HTG-AP, and SVT, emphasizing the complex interplay between metabolic dysfunction, pancreatic inflammation, and thrombosis. Early recognition and prompt treatment with intravenous insulin, supportive care, and individualized anticoagulation were associated with a favorable clinical outcome. Further prospective studies are needed to better define the role and optimal duration of anticoagulation in pancreatitis-associated SVT.

## References

[1] Lizzo JM, Goyal A, Kaur J (2026). Adult diabetic ketoacidosis. StatPearls.

[2] Tiperneni R, Padappayil RP, Mohan G, Patton C (2022). Diabetic ketoacidosis and hypertriglyceridemia induced pancreatitis: can the perfect storm happen twice?. J Community Hosp Intern Med Perspect.

[3] Patlori S, Deepak P, Rao NV (2025). Long-term outcomes of splanchnic venous thrombosis in acute pancreatitis. Pancreas.

[4] Yang AL, McNabb-Baltar J (2020). Hypertriglyceridemia and acute pancreatitis. Pancreatology.

[5] Faheem B, Singh B, Ashkar H, Gupta S, Kaur P, Maroules M (2022). Tetrad of DKA, hypertriglyceridemia induced pancreatitis and splenic vein thrombosis. J Community Hosp Intern Med Perspect.

[6] Hahn SJ, Park JH, Lee JH, Lee JK, Kim KA (2010). Severe hypertriglyceridemia in diabetic ketoacidosis accompanied by acute pancreatitis: case report. J Korean Med Sci.

[7] Fulop M, Eder H (1990). Severe hypertriglyceridemia in diabetic ketosis. Am J Med Sci.

[8] Hansen SE, Varbo A, Nordestgaard BG, Langsted A (2023). Hypertriglyceridemia-associated pancreatitis: New concepts and potential mechanisms. Clin Chem.

[9] Kiss L, Fűr G, Pisipati S, Rajalingamgari P, Ewald N, Singh V, Rakonczay Z Jr (2023). Mechanisms linking hypertriglyceridemia to acute pancreatitis. Acta Physiol (Oxf).

[10] Liu J, Gong H, Chen X, Tang C, Huang L (2024). A narrative review of acute pancreatitis-induced splanchnic vein thrombosis: from pathogenesis to clinical management. Scand J Gastroenterol.

[11] Borbély RZ, Szalai EÁ, Philip BM (2024). The risk of developing splanchnic vein thrombosis in acute pancreatitis increases 3 days after symptom onset: A systematic review and meta-analysis. United European Gastroenterol J.

[12] Gotto A, Lieberman M, Pochapin M (2014). Gastric variceal bleeding due to pancreatitis-induced splenic vein thrombosis. BMJ Case Rep.

[13] Butler JR, Eckert GJ, Zyromski NJ, Leonardi MJ, Lillemoe KD, Howard TJ (2011). Natural history of pancreatitis-induced splenic vein thrombosis: a systematic review and meta-analysis of its incidence and rate of gastrointestinal bleeding. HPB (Oxford).

[14] Barnum KJ, Patell R, Berry J, Bauer KA (2025). Splanchnic vein thrombosis: management for the thrombosis specialist. J Thromb Haemost.

[15] Hostiuc M, Negoi I (2025). Etiology and risk factors for splanchnic vein thrombosis in non-cirrhotic, non-neoplastic patients: a narrative review. Medicina (Kaunas).

[16] How J, Zhou A, Oh ST (2017). Splanchnic vein thrombosis in myeloproliferative neoplasms: pathophysiology and molecular mechanisms of disease. Ther Adv Hematol.

[17] Umpierrez GE, Davis GM, ElSayed NA (2024). Hyperglycaemic crises in adults with diabetes: a consensus report. Diabetologia.

[18] Subramanian S, Soran H, Sikora Kessler A, McClain MR, Eckel RH, Rosenson RS (2025). Prevention and treatment of hypertriglyceridemia-mediated acute pancreatitis: A narrative review. Eur J Intern Med.

[19] Al-Ismail M, Promi T, Hetai R, Ahmed S, Awaisu A (2026). Efficacy and safety of pharmacological and procedural interventions in the management of hypertriglyceridemia-induced acute pancreatitis: a systematic review. Saudi Pharm J.

[20] Fang C, Ding Y, Wang X, Teng X (2025). Metabolic disturbances in acute pancreatitis: mechanisms and therapeutic implications. Front Endocrinol (Lausanne).

[21] Scott M, Ghazanfar M, Windsor J, Ramsay G, Bekheit M (2025). The management of splanchnic vein thrombosis in acute pancreatitis: a global DELPHI consensus study. HPB (Oxford).

[22] Yin Y, Wang L, Gao F, Liu L, Qi X (2023). Anticoagulation therapy for splanchnic vein thrombosis associated with acute pancreatitis: a systematic review and meta-analysis. Clin Appl Thromb Hemost.

[23] Harris S, Nadkarni NA, Naina HV, Vege SS (2013). Splanchnic vein thrombosis in acute pancreatitis: a single-center experience. Pancreas.

[24] Custo S, Tabone E, Aquilina A, Gatt A, Riva N (2024). Splanchnic vein thrombosis: The state-of-the-art on anticoagulant treatment. Hamostaseologie.

[25] Öğütmen Koç D, Bengi G, Gül Ö (2024). Turkish Society of Gastroenterology: Pancreas Working Group, Acute Pancreatitis Committee consensus report. Turk J Gastroenterol.

